# Antifungal Action of *Arabidopsis thaliana* TCP21 via Induction of Oxidative Stress and Apoptosis

**DOI:** 10.3390/antiox12091767

**Published:** 2023-09-15

**Authors:** Seong-Cheol Park, A-Mi Yoon, Young-Min Kim, Min-Young Lee, Jung Ro Lee

**Affiliations:** 1 Department of Chemical Engineering, Sunchon National University, Suncheon 57922, Republic of Korea; schpark9@gnu.ac.kr (S.-C.P.); metingym@scnu.ac.kr (Y.-M.K.); 2 LMO Team, National Institute of Ecology (NIE), Seocheon 33657, Republic of Korea; pinus457@nie.re.kr; 3 Division of Life Sciences, Jeonbuk National University, Jeonju 54896, Republic of Korea; 4 Department of Clinical Laboratory Science, Daejeon Health Institute of Technology, Daejeon 34504, Republic of Korea; mylee365@hit.ac.kr; 5 Department of Biochemistry and Biophysics, Texas A&M University, College Station, TX 77843, USA

**Keywords:** antifungal protein, TCP, oxidative stress, reactive oxygen species, apoptosis

## Abstract

The realm of antimicrobial proteins in plants is extensive but remains relatively uncharted. Understanding the mechanisms underlying the action of plant antifungal proteins (AFPs) holds promise for antifungal strategies. This study aimed to bridge this knowledge gap by comprehensively screening *Arabidopsis thaliana* species to identify novel AFPs. Using MALDI-TOF analysis, we identified a member of the TEOSINTE BRANCHED1/CYCLOIDEA/PROLIFERATING CELL FACTOR1 (TCP) family of transcription factors as a novel AFP, *A. thaliana* TCP21 (AtTCP21; accession number NP_196450). Bacterially purified recombinant AtTCP21 inhibited the growth of various pathogenic fungal cells. AtTCP21 was more potent than melittin, a well-known AFP, in combating *Colletotrichum gloeosporioides*. Growth inhibition assays against various fungal pathogens and yeasts confirmed the pH-dependent antimicrobial activity of AtTCP21. Without inducing any membrane alterations, AtTCP21 penetrates the fungal cell wall and membrane, where it instigates a repressive milieu for fungal cell growth by generating intracellular reactive oxygen species and mitochondrial superoxides; resulting in morphological changes and apoptosis. Our findings demonstrate the redox-regulating effects of AtTCP21 and point to its potential as an antimicrobial agent.

## 1. Introduction

Antimicrobial resistance is a growing global health crisis, with an increasing number of pathogenic bacteria and fungi becoming resistant to existing antimicrobial agents [1]. Therefore, the discovery and development of new antimicrobial agents is a priority in the fight against infectious diseases [2]. Plant proteins are promising antimicrobial agents, as many have been shown to possess potent antibacterial and antifungal activities [3,4,5,6].

The TEOSINTE BRANCHED1/CYCLOIDEA/-PROLIFERATING CELL FACTOR1 (TCP) gene family regulates plant growth and development. It was given this name due to its four unrelated proteins: teosinte branched1 (TB1), cycloidea (CYC), and proliferating cell nuclear antigen factors (PCF1 and PCF2). The TB1 gene is a critical determinant of robust apical dominance in cultivated maize [7] while CYC plays a crucial role in regulating bilateral symmetry in the flowers of Antirrhinum [8]. PCF1 and PCF2 are factors that bind to the promoter of the rice proliferating cell nuclear antigen (*PCNA*) gene [9]. This gene encodes a protein involved in DNA replication and repair, chromatin structure maintenance, chromosome segregation, and cell-cycle progression. The TCP family members have a conserved basic helix–loop–helix (bHLH) structure comprising approximately 58–62 amino acids involved in DNA binding, protein–protein interactions, and the intranuclear localization of proteins. The TCP family can be categorized into Class I and II proteins [10,11]. Class I proteins regulate various aspects of plant growth and development, such as stem cell proliferation and organ formation, while Class II proteins regulate light-related responses [10,11,12]. Proteins of the two classes can be differentiated based on variations within their TCP domains: Class I (referred to as PCF or TCP-P) and Class II (known as the TCP-C class) [9,11,13]. Class I includes rice PCF1 and PCF2, while Class II encompasses CYC and TB1. The primary distinguishing factor between Class I and II proteins is a four-amino acid deletion within the TCP domain, which is unique to Class I but absent in Class II. The landscape of the TCP family proteins has expanded significantly, requiring a wide-angle lens to encompass this continuously growing family [10,12]. However, some TCP genes in the root and leaf tissues of bananas are highly expressed in response to *Fusarium oxysporum* f. sp. *cubense* infection, which is known as a major threat to banana production [14]. Another study reported that TCP29 overexpression increased the susceptibility of transgenic Arabidopsis to *Botrytis cinerea* [15].

Despite the importance of the TCP family proteins in plant biology and immunity, much remains to be learned about their potential properties [16]. This study aimed to assess the antimicrobial activity of *Arabidopsis thaliana* TCP21, a Class I TCP protein, against various fungal strains. Our findings provide crucial information on the antimicrobial properties of TCP21 via the induction of reactive oxygen species (ROS) and apoptosis and contribute to a better understanding of the biological functions of the TCP family of proteins. Additionally, the results of this study highlight the potential of TCP family proteins as targets for antimicrobial drug development and could have implications for new strategies against infectious diseases.

## 2. Materials and Methods

### 2.1. Fungal Cells and Growth Conditions

*Candida albicans* (KCTC 7270), *C. tropicalis* (KCTC 7221), *Trichosporon beigelii* (KCTC 7707), *Colletotrichum gloeosporioides* (KCTC 6169), *Fusarium graminearum* (KCTC 16656), *F. moniliforme* (KCTC 6149), *F. oxysporum* (KCTC 16909), *F. solani* (KCTC 6326), *Trichoderma harzianum* (KCTC 6043), and *T. viride* (KCTC 16992) were obtained from the Korea Collection for Type Cultures (KCTC, Jeongup-si, Jeollabuk-do, Korea). Mold fungi were grown in potato dextrose (PD; Difco, Sparks, MD, USA) agar for 5 days, and yeast fungi were incubated in yeast extract–peptone–dextrose (YPD, Difco) agar or medium.

### 2.2. Purification and Characterization of Recombinant AtTCP21

AtTCP21 DNA was isolated from an Arabidopsis cDNA library by polymerase chain reaction (PCR), and the accuracy of the cDNA constructs was verified by sequencing analysis. The AtTCP21 protein was produced in *Escherichia coli* by inserting the *AtTCP21* gene into the pET28a expression vector. The pET28a-AtTCP21 plasmid was introduced into *E. coli* BL21(DE3) cells and cultured in LB medium. The Histidine-tagged AtTCP21 protein was then purified using a nickel–nitrilotriacetic acid (Ni-NTA) resin (Qiagen GmbH, Hilden, Germany) and eluted in imidazole (70 or 250 mM)-containing buffer. The FPLC system was utilized at 25 °C and a 0.6 mL/min flow rate for column equilibration, employing 25 mM HEPES buffer (pH 7.2)-containing 150 mM NaCl (Buffer A). Size exclusion chromatography (SEC) was subsequently performed, and the SEC column was calibrated using the Gel Filtration Standard (Bio-Rad, Hercules, CA, USA), which included thyroglobulin (670 kDa), globulin (158 kDa), ovalbumin (44 kDa), myoglobin (17 kDa), and vitamin B12 (1.35 kDa) as references [17,18]. Protein purity was evaluated via SDS-PAGE as described previously [17]. The purified recombinant protein was dialyzed against buffer A and subsequently used for biochemical investigation.

### 2.3. Transmission Electron Microscopy (TEM)

Purified AtTCP21 protein was dropped onto a carbon-coated copper grid that was glow-discharged into the air using a plasma cleaner (Harrick Plasma, Ithaca, NY, USA). After 3 min, the liquid solution was removed by touching the filter paper and the sample was negatively stained with 2% (*w/v*) uranyl acetate. The grids were examined using a 200 kV FEI Tecnai 20 TEM (FEI, Hillsboro, OR, USA) equipped with a Gatan CCD camera.

### 2.4. Antifungal Assay

The antifungal activity of AtTCP21 was measured spectrophotometrically via a microtiter assay. Yeast fungi were cultured overnight in YPD broth, and mold spores were collected from 4-day-old cultures grown on PD agar plates using 0.08% Triton X-100. Fungal suspensions in Buffer A, or 25 mM MES buffer with 150 mM NaCl (pH 5.5) containing 20% of the appropriate media (2 × 10^4^ cells or spores/mL), were added to serially diluted AtTCP21 proteins and melittin peptides in 96-well plates [19]. After 24 h incubation at 28 °C, 10 μL of cells was dropped on PDA agar, and cell/mycelial growth was measured at 595 nm using an ELISA reader (Molecular Devices M5, Sunnyvale, CA, USA). The inhibitory concentration 50 (IC_50_) was defined as the concentration of an antifungal agent that reduces the growth by 50%.

### 2.5. SYTOX Green Uptake Assay

*C. gloeosporioides* and *F. graminearum* conidia collected from mycelial cells were mixed with AtTCP21 (IC_50_ concentration) or H_2_O_2_ (1 mM). After 4 h, cells were incubated with SYTOX Green dye (Invitrogen, Eugene, OR, USA; final concentration to 0.2 μM) for 15 min in the dark. The stained cells were observed under a fluorescence microscope (OPTINIT KCS3-160S, Korea Lab Tech, Namyangju, Republic of Korea), and the shift in fluorescence was analyzed using flow cytometry (Attune NxT, ThermoFisher, Seoul, Republic of Korea).

### 2.6. Live/Dead Cell Assay

Conidial cells of *C. gloeosporioides* and *F. graminearum* were incubated with AtTCP21 or melittin (IC_50_ concentration) for 4 h. Cells were washed with buffer A, followed by the addition of 10 μM FUN-1 (2-chloro-4-(2,3-dihydro-3-methyl-(benzo-1,3-thiazol-2-yl)-methylidene)-1-phenylquinolinium iodide) and 25 μM Calcofluor white M2R dyes. After 30 h of incubation in the dark, the cells were observed under a fluorescence microscope.

### 2.7. Confocal Laser Scanning Microscopy (CLSM)

AtTCP21 and melittin were labeled by adding Flamma^®^ 675-NHS (BioActs, Incheon, Republic of Korea), a fluorescent dye solution, to the proteins in buffer A at a molar ratio of 1:1. After 2 h of incubation, the mixtures were dialyzed against PBS for 48 h to remove any unbound fluorescent dye. The labeled protein samples were incubated with *C. gloeosporioides* cells at 28 °C for 4 h. The fungal cells were washed thrice with buffer A, mounted on a cover glass in 50% glycerol and 0.1% n-propyl gallate solution, and examined under a CLSM (A1R HD 25, Nikon, Japan) [19].

### 2.8. Reactive Oxygen Species (ROS) Assessment

Intracellular ROS generation was analyzed using 2′,7′-dichlorodihydrofluorescein diacetate (DCF-DA) (Invitrogen, Eugene, OR, USA). Conidia of *C. gloeosporioides*, *F. graminearum*, or *F. solani* (5 × 10^4^ cells/mL) were treated with AtTCP21 (IC_50_ concentration) or H_2_O_2_ (1 mM) in buffer A containing 10% PDB for 6 h at 25 °C. The conidial cells were stained with DCF-DA (10 μM) for 30 min and their fluorescence was measured at an excitation of 495 nm and emission of 525 nm using a spectrofluorometer (Perkin-Elmer LS55, Mid Glamorgan, UK). To evaluate the ROS levels during hyphal growth, conidia were incubated at 25 °C for 12 h, which allowed them to grow into hyphae, following which AtTCP21 or H_2_O_2_ was added. After incubating for another 4 h at 25 °C, the cells were stained with DCF-DA for 30 min and observed under a fluorescence microscope [20]. 

### 2.9. Quantification of Reduced Glutathione (GSH)

The GSH content was measured using Ellman’s assay [21]. Conidia of *C. gloeosporioides* (5 × 10^4^ cells/mL) were incubated with AtTCP21 or melittin (IC_50_ concentration) for 6 h at 25 °C and washed with cold buffer A. The cells were lysed via freeze/thawing (seven cycles), centrifuged at 6000× *g* for 10 min, and the supernatants were collected. Next, 5,5-dithiobis(2-nitrobenzoic acid) solution was added to the supernatants and incubated for 30 min in the dark at 37 °C. The absorbance was measured at 412 nm using a spectrofluorometer (Perkin-Elmer LS55).

### 2.10. Measurement of Mitochondrial Superoxide and Membrane Potential

MitoSOX^®^ Red probe (Invitrogen, Eugene, OR, USA) was used to measure mitochondrial superoxide (SOX) production in fungal cells. Fungal conidia were treated with AtTCP21 (IC_50_ concentration) or H_2_O_2_ (1 mM) for 6 h and stained according to the manufacturer’s protocol. Cells were analyzed via fluorescence microscopy and flow cytometry (Attune NxT) [20].

### 2.11. Cytochrome c Release Assay

*C. gloeosporioides* conidial cells (5 × 10^5^ cells/mL) treated with AtTCP21 or melittin (IC_50_ concentration) for 4 h were sonicated in 50 mM Tris buffer (pH 7.4) containing 2 mM EDTA and 1 mM phenylmethylsulfonyl fluoride. After centrifugation, the supernatants were stored at 4 °C. Mitochondria were collected using a mitochondria isolation kit (Abcam, Cambridge, MA, USA). Ascorbic acid of 500 mg/mL was added as a reducing agent for cytochrome c, and absorbance was measured at 550 nm (Molecular Devices M5).

### 2.12. Apoptosis Assay

Fungal cell apoptosis was assessed via flow cytometry with propidium iodide (PI, Invitrogen) and Annexin V-Flamma 488 dyes (V-488, Bioacts). *C. gloeosporioides* conidia were incubated with AtTCP21 or melittin (IC_50_ concentration) for 8 h and then washed with buffer A. V-488 and PI were added to the suspensions as per the manufacturer’s protocol. The percentages of necrotic and apoptotic cells were analyzed via flow cytometry (Attune NxT) [19].

### 2.13. Caspase 3/7 Activation Assay

*C. gloeosporioides* conidial cells treated with AtTCP21 or melittin were incubated with CellEvent^TM^ caspase-3/7 Green detection reagent and SYTOX^TM^ AADvanced^TM^ Dead cell stain as per the manufacturer’s protocol (Invitrogen). The stained cells were analyzed via flow cytometry (Attune NxT).

### 2.14. Nuclear Staining with Hoechst 33342

*C. gloeosporioides* conidial cells treated with AtTCP21 or melittin (IC_50_ concentration, 6 h) were stained with Hoechst 33342 (Invitrogen) dye and incubated for 20 min at 25 °C. The stained cells were observed under a fluorescence microscope and the blue fluorescence intensity was recorded at the same level.

### 2.15. Scanning Electron Microscopy (SEM)

*C. gloeosporioides* conidial cells (5 × 10^5^ cells/mL) treated with AtTCP21 or melittin (IC_50_ concentration) for 12 h were fixed with 2% glutaraldehyde (*v/v*) in 0.2 M HEPES buffer (pH 8.0) overnight at 4 °C. The fixed cells were postfixed in 1% osmium tetroxide (*w*/*v*; Electron Microscopy Sciences, Hatfield, PA, USA) for 1 h, followed by dehydration with OTTIX Shaper (Diapath S.p.A, Bergamo, Italy). The cells were chemically dried using hexamethyldisilazane sputter-coated with gold–palladium and were observed via SEM (JSM-7100F; JEOL, Ltd., Tokyo, Japan) [19].

### 2.16. Statistical Analysis

All experiments were performed in triplicate, and the data have been presented as mean ± SD. Statistical analyses were performed using Excel software (Microsoft Office 2016). The results were compared using a one-way ANOVA test, and findings with *p* < 0.05 and 0.01 were considered significant.

## 3. Results and Discussion

### 3.1. Purification and Identification of an Arabidopsis TCP Protein with Antifungal Activity

**Figure 1:**
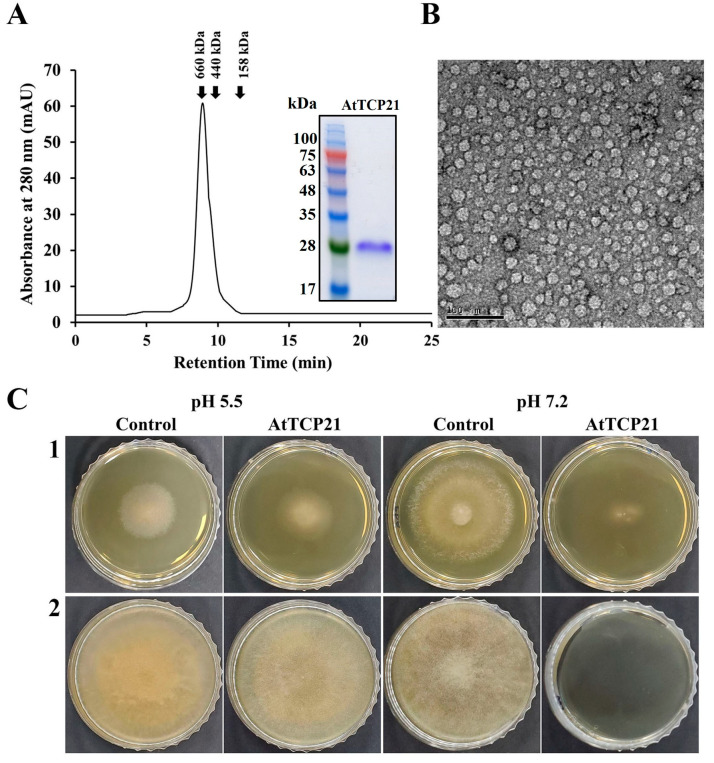
Characterization of recombinant AtTCP21 based on (**A**) size distribution, (**B**) structure, and (**C**) antifungal activity. (**A**) Size exclusion chromatography of recombinant AtTCP21 on Superdex S-200 column. Bacterially expressed recombinant AtTCP21 protein was resolved with 12% SDS-PAGE (inset). (**B**) Purified AtTCP21 was observed under a transmission electron microscope. (**C**) Inhibitory action of AtTCP21 on the fungal growth. The (1) *C. gloeosporioides* and (2) *F. oxysporum* spores were incubated with two-fold diluted concentrations of AtTCP21 at pH 5.5 or 7.2 for 24 h. The treated spores (20 µL) were plated on agar plates and incubated for 48 h.

Plants have evolved an array of potent defense molecules, including antifungal proteins (AFPs), phytoalexins, and other proteins, in response to a multitude of pathogenic fungi during their growth in the natural environment [21,22,23,24]. We identified and isolated novel proteins from *A. thaliana* species using two-dimensional PAGE to elucidate the defense mechanisms of plant proteins. Using MALDI-TOF techniques, we successfully isolated a TCP transcription factor that perfectly corresponded to the AtTCP21 protein of *A. thaliana* (accession number NP_196450). To investigate the physiological functions of the AtTCP21 protein, we expressed the full-length AtTCP21 in *E. coli* BL21(DE3). Subsequently, the recombinant AtTCP21 protein was purified using affinity chromatography. SEC analysis confirmed the successful elution of AtTCP21 (Figure 1A). Furthermore, TEM analysis of the negatively stained recombinant AtTCP21 protein revealed planar low oligomers and their assembled globular three-dimensional high-oligomer structures (Figure 1B).

We further assessed AtTCP21 protein activity under different pH conditions. As shown in Figure 1C, the protein exhibited potent antifungal efficacy against both *C. gloeosporioides* (1) and *F. oxysporum* (2) strains, indicated by the inhibition of mycelial growth in mold fungi. Notably, the antifungal activity was higher at pH 7.2 than at pH 5.5. Since the cytosolic pH of Arabidopsis protoplasts is 7.3 ± 0.1 [25], the antifungal activity of AtTCP21 is expected to be robust under the physiological conditions of Arabidopsis plants.

The antifungal activity of recombinant AtTCP21 was evaluated using a microtiter plate assay. AtTCP21 protein impeded the growth of the tested mold and yeast fungi at IC_50_ concentrations ranging from 3.1 to 100 μg/mL (Table 1). However, its antifungal activity was lower than melittin, a control antifungal peptide effective against most of the examined fungal strains, except *C. gloeosporioides*. Remarkably, IC_50_ for AtTCP21 was about fourfold higher than that of melittin against *C. gloeosporioides*, a fungus that causes bitter rot in a variety of crops worldwide, including avocado, citrus, coffee, eggplant, papaya, tomato, sweet pepper, and yam, resulting in enormous economic losses owing to pre- and post-harvest damage.

**Figure 2:**
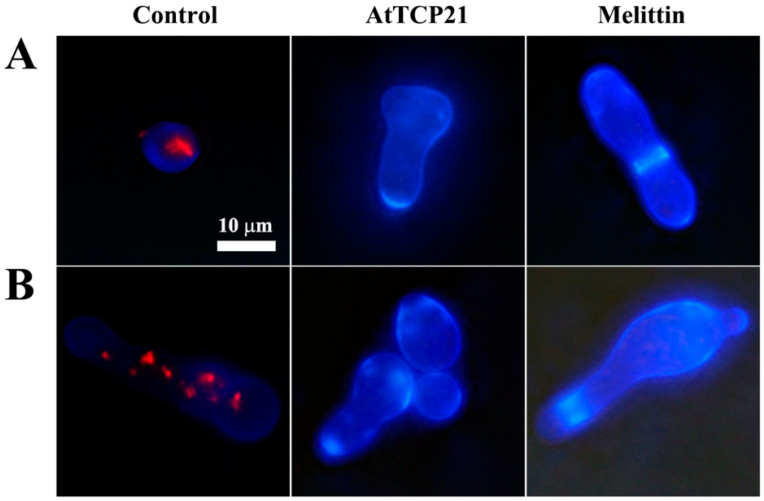
The effect of AtTCP21 and melittin on cell viability. (**A**) *C. gloeosporioides* and (**B**) *F. graminearum* cells treated with AtTCP21 or melittin were stained using the live/dead cell assay. FUN-1 dye stains the intracellular intravacuolar structures, and Calcofluor white M2R labels cell-wall chitin, emitting red-orange and blue fluorescence, respectively.

To investigate whether the antifungal activity of AtTCP21 was due to a fungistatic or fungicidal effect, *C. gloeosporioides* cells treated with the proteins were stained with FUN-1 and Calcofluor white M2R dyes. FUN-1 stains metabolically active cells and emits an orange-red fluorescence. Calcofluor white M2R is a non-specific dye that can bind with fungal cellulose and chitin. As shown in Figure 2, strong red fluorescence was detected in the cytosol of the untreated cells, while no fluorescence was detected in the AtTCP21 and melittin-treated cells, indicating they were dead cells. Moreover, the increase in cell size suggested physiological changes potentially associated with cell death.

### 3.2. Cellular Distribution of AtTCP21 in Fungal Cells

**Figure 3:**
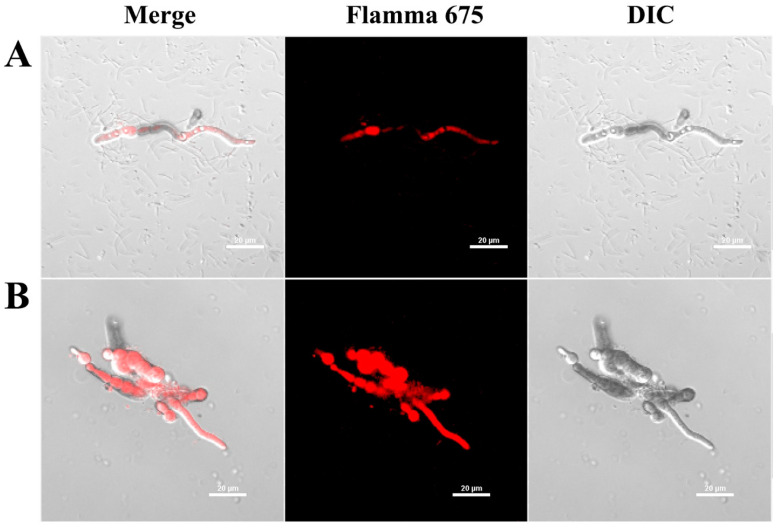
Cellular distribution of Flamma 675-labeled (**A**) AtTCP21 and (**B**) melittin in *C. gloeosporioides*. After incubation of dye-labeled proteins with *C. gloeosporioides* for 4 h, the washed and fixed fungal cells were observed under confocal laser scanning microscopy (CLSM).

To elucidate whether the AtTCP21 protein acts on the cell wall/membrane or undergoes translocation into the cytoplasm with regard to its antifungal activity, we investigated the cellular distribution of the Flamma 675-labeled AtTCP21 in *C. gloeosporioides* cells using confocal laser scanning microscopy (CLSM) (Figure 3). *C. gloeosporioides* cells treated with Flamma 675-labeled AtTCP21 for 4 h showed pronounced protein accumulation within the cytosol, which was evident in both the spores and hyphae. However, melittin-treated cells showed fluorescence on the cell surface and in the cytosol. This pattern of fluorescence distribution strongly suggests that AtTCP21 effectively traverses the cell membrane and enters the cytoplasm. Indeed, while the compositions of fungal cell walls can vary, fungal cells have the capability to transport macromolecules into their cytosol via processes such as endocytosis. This mechanism allows them to internalize and interact with external molecules, which can have important implications for their physiology and interactions with their environment.

### 3.3. Cellular Uptake of AtTCP21 without Alteration of Membrane Integrity 

Several mechanisms have been described for the cytosolic translocation of AFPs and other proteins across fungal cell walls and membranes. These include direct penetration, vacuolar localization and expansion, limited disruption of the plasma membrane, formation of transition pores, and endocytosis [26,27]. To ascertain whether AtTCP21 translocation into the cytosol was facilitated by membrane damage, we used a membrane-impermeable probe, SYTOX Green, which emits green fluorescence upon binding to nucleic acids and can only infiltrate cells when the membrane integrity is compromised or when antifungal agents create pores.

**Figure 4:**
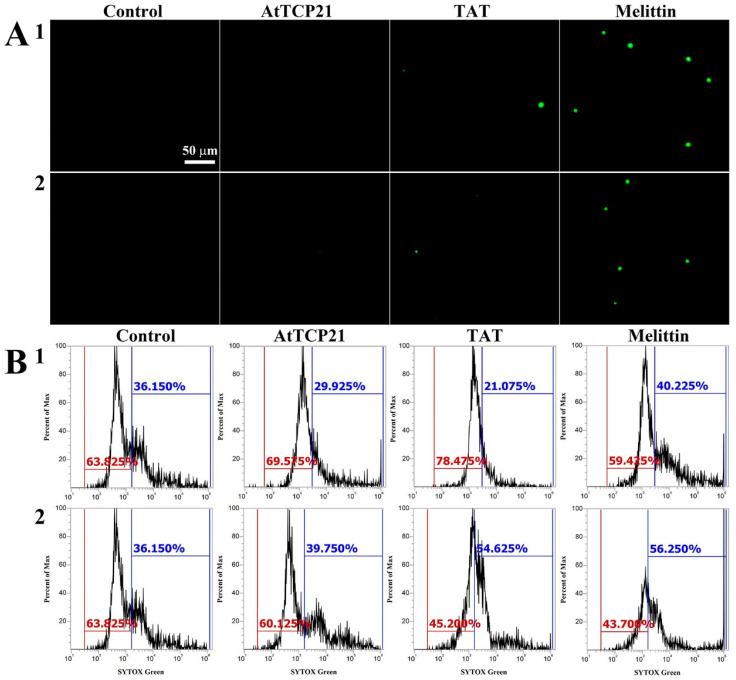
SYTOX Green uptake in (1) *C. gloeosporioides* and (2) *F. graminearum* conidial cells. AtTCP21, TAT, and melittin proteins were incubated with fungal conidia for 4 h, followed by the addition of SYTOX Green dye. After further incubation for 15 min, cells were (**A**) observed under a fluorescence microscope or (**B**) analyzed via flow cytometry.

We used fluorescence microscopy (Figure 4A) and flow cytometry (Figure 4B) to investigate any AtTCP21-induced changes in membrane integrity. Notably, while melittin-treated *C. gloeosporioides* and *F. graminearum* cells showed green fluorescence, AtTCP21-treated cells did not. TAT, a cell-penetrating peptide known to form transient pores when introduced into cells, was a positive control for SYTOX Green influx. The findings in Figure 4 suggest that AtTCP21 enters the cell without damaging the cell membrane. However, the cell-penetrating action of the TAT peptide is instantaneously exhibited via self-created pores, indicating some SYTOX Green uptake. Additionally, melittin causes damage to the fungal membrane and enters the cytosol. These results suggest that the TAT and melittin, except AtTCP21, alter membrane integrity to pass through the cell wall and accumulate in the cytoplasm of fungal cells.

### 3.4. AtTCP21-Induced Intracellular ROS Generation

**Figure 5:**
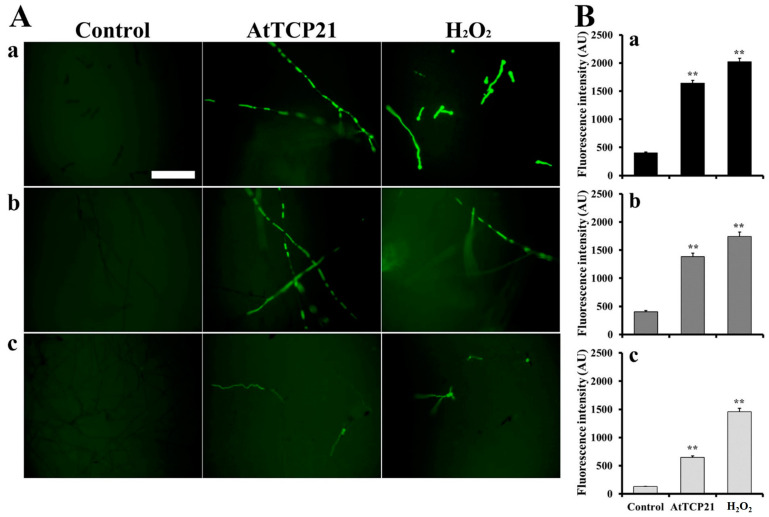
Generation of intracellular ROS in response to AtTCP21 in the hyphae and conidia of (**a**) *C. gloeosporioides,* (**b**) *F. graminearum*, and (**c**) *F. solani* cells. Panel (**B**) shows the quantification of the fluorescence data (**A**). Statistical significance was analyzed via the one-way ANOVA test, compared to control (** *p* < 0.01). The bar is 100 μm.

With regard to the antifungal activity of proteins entered into fungal cells, various events can occur, including ROS generation, inhibition of protein synthesis, and binding with nucleic acids. To determine the AtTCP21-induced intracellular responses in fungal cells, we examined ROS generation using H2DCF-DA, a fluorescent probe sensitive to ROS, including hydrogen peroxide, hydroxyl radicals, and peroxynitrite. Fluorescence was evident in the hyphae of *C. gloeosporioides*, *F. graminearum*, and *F. solani* cells treated with AtTCP21 (IC_50_ concentration) or H_2_O_2_ (1 mM), indicating the accumulation of excessive ROS (Figure 5A). However, the number of fluorescent cells in conidia was higher following H_2_O_2_ treatment than AtTCP21 treatment, consistent with the observations in Figure 5A (Figure 5B). The results suggested that the AtTCP21 protein exhibits an antifungal effect via ROS generation in the AtTCP21-treated fungal cells.

**Figure 6:**
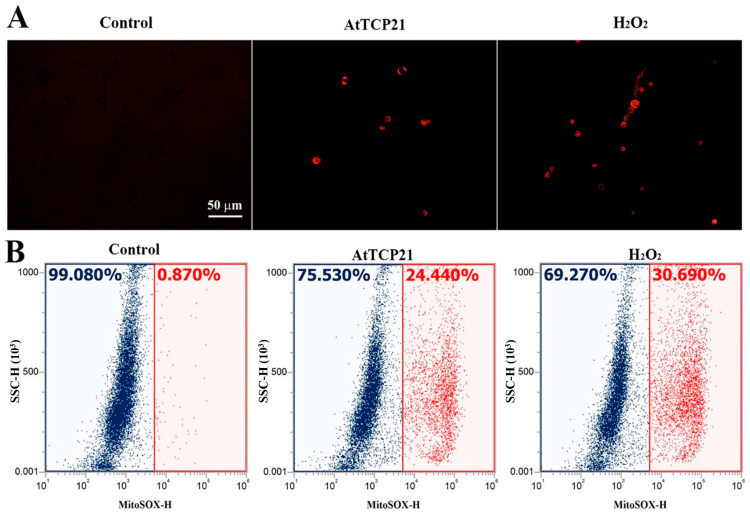
AtTCP21-induced production of mitochondrial superoxide in *C. gloeosporioides* cells. After incubation of AtTCP21 (IC_50_ concentration) or H_2_O_2_ (1 mM) for 6 h in conidial cells, they were observed under fluorescence microscope (**A**) and analyzed using flow cytometry (**B**).

Mitochondria play a critical role in regulating cellular apoptosis and excessive ROS generation. They have the capacity to trigger the opening of the permeable transition pore, the release of cytochrome c, and the activation of the caspase system, ultimately resulting in apoptosis. We focused on AtTCP21’s action in mitochondria, as we hypothesized that AtTCP21 would struggle to generate ROS in fungal cells without interacting with other proteins or organelles. To determine if AtTCP21 primarily induces mitochondrial ROS in fungal cells, we used MitoSOX Red, a fluoroprobe that detects mitochondrial superoxide (SOX). Fluorescence microscopy revealed a notable increase in MitoSOX Red fluorescence in *C. gloeosporioides* cells treated with AtTCP21 and H_2_O_2_ (Figure 6A). Flow cytometric analysis of cells treated with AtTCP21 and H_2_O_2_ showed a 24.44% and 30.69% shift in the red fluorescence, respectively. Mitochondria regulate cell death and ROS production by initiating permeable transition pore openings, cytochrome c release, and activation of caspase cascades, ultimately culminating in apoptosis. Our observations confirm the involvement of increased mitochondrial ROS generation in AtTCP21-induced apoptosis of fungal cells. 

**Figure 7:**
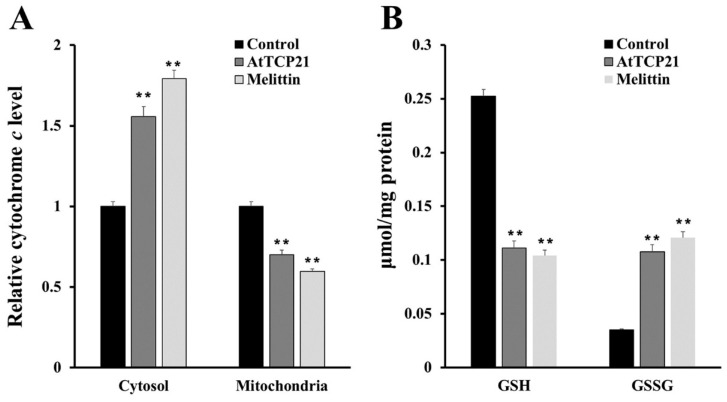
Quantification of cytochrome c release (**A**), levels of reduced GSH, and oxidized GSSG (**B**) in AtTCP21- and melittin-treated *C. gloeosporioides* cells (** *p* < 0.01).

To further detect mitochondrial dysfunction, we quantified cytochrome *c* release in the cytosol and mitochondria of AtTCP21-treated *C. gloeosporioides* cells (Figure 7A). In addition, intracellular levels of GSH and GSSG (efficient antioxidant molecules) were also assessed (Figure 7B). AtTCP21 treatment increased the relative cytochrome c levels in the cytosol while decreasing them in the mitochondria (Figure 7A). The intracellular levels of GSH dramatically decreased in response to AtTCP21 and melittin, whereas the GSSG levels significantly increased (Figure 7B). These findings indicate that AtTCP21 causes mitochondrial dysfunction to alter the cellular redox state and induce apoptosis.

### 3.5. AtTCP21 Induces Apoptosis and Activates Caspase 3/7 

**Figure 8:**
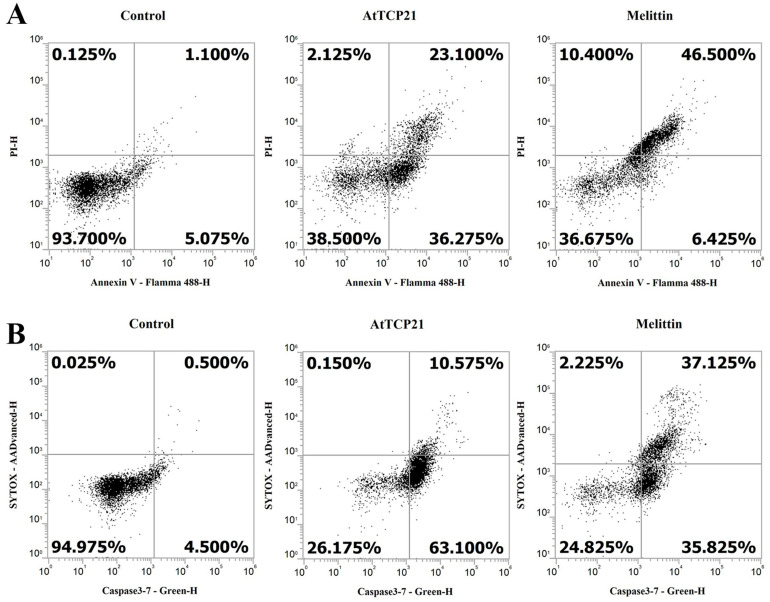
Induction of apoptosis and caspase 3/7 activation in AtTCP21-treated *C. gloeosporioides* cells. *C. gloeosporioides* conidia were incubated with AtTCP21 or melittin at IC_50_ concentration for 8 h and stained with Annexin V-Flamma 488/PI (**A**) or caspase-3-7/SYTOX AADvanced dyes (**B**), flowed via flow cytometry analysis.

Loss of plasma membrane asymmetry is one of the early morphological characteristics of apoptosis. Under normal conditions, phosphatidylserine (PS) is present on the inner leaflet of the plasma membrane. However, in apoptotic cells, PS is externalized and is present on the outer leaflet of the membrane. The exposed PS can, therefore, bind with high affinity to annexin V, a Ca^2+^-dependent phospholipid-binding protein [17,28,29]. Propidium iodide (PI) is an impermeable dye in live and apoptotic cells but can stain necrotic dead cells in red fluorescence by binding to nucleic acids. In *C. gloeosporioides* cells, AtTCP21 induced 59.4% apoptosis and 2.1% necrosis, whereas melittin induced 52.9% apoptosis and 10.4% necrosis (Figure 8A). Another distinctive feature of the early stages of apoptosis is the activation of caspase 3/7, a key mediator of apoptosis-related mitochondrial events [30]. To detect activated caspase 3 and 7 in apoptotic cells, we used a cell-permeant fluorogenic substrate (DEVD peptide). SYTOX AADvanced dye is impermeable in live cells but can stain nucleic acids through penetration into cells with compromised plasma membranes. As shown in Figure 8B, AtTCP21 induced 63.1% apoptosis and 10.6% necrosis. *C. gloeosporioides* treated with melittin showed similar percentages of apoptotic and necrotic cells. These results suggest that the leading cause of antifungal activity of AtTCP21 is apoptosis induced by ROS generation, and that melittin exhibits antifungal activity through the dual action of cell membrane destruction and ROS generation.

**Figure 9:**
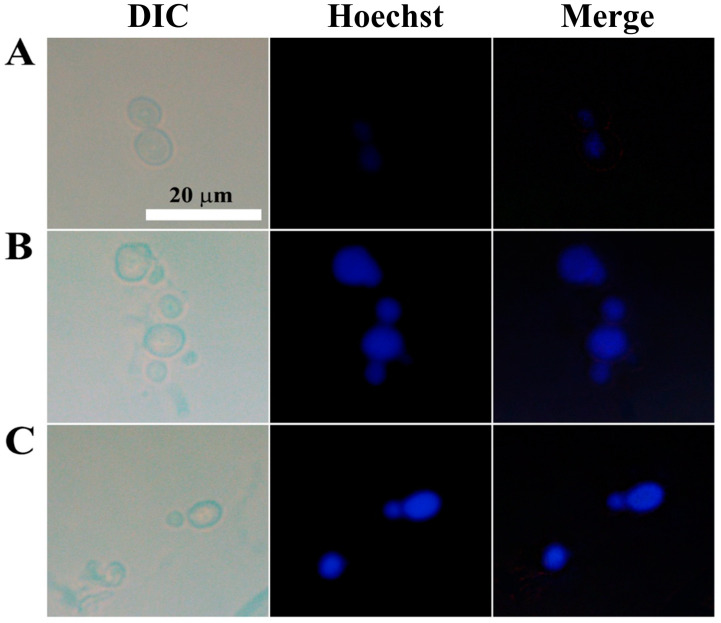
Nuclear staining of *C. gloeosporioides* conidial cells that were (**A**) untreated, (**B**) AtTCP21- or (**C**) melittin-treated. After incubating with AtTCP21 or melittin (IC_50_ concentration, 6 h), the cells were stained with Hoechst 33342 dye for 20 min and observed under a fluorescence microscope.

To detect chromatin condensation, another morphological hallmark of apoptosis, we stained *C. gloeosporioides* with Hoechst 33342, a cell-permeable dye that stains the condensed chromatin of apoptotic cells more brightly than normal cells. While the control cells showed dim blue fluorescence, AtTCP21- and melittin-treated cells emitted strong fluorescence (Figure 9). These findings further prove that AtTCP21’s antifungal activity is mediated by apoptosis.

### 3.6. Morphological Alterations Induced by AtTCP21 in C. gloeosporioides

**Figure 10:**
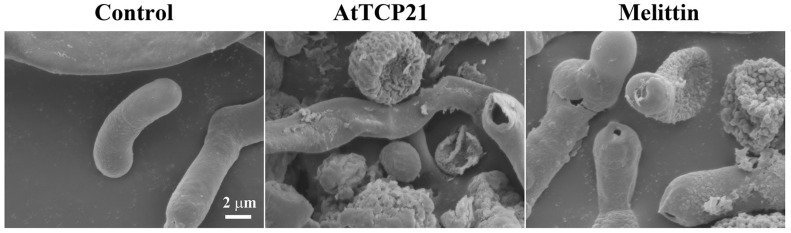
Antifungal effects of AtTCP21 and melittin in *C. gloeosporioides* cells.

*C. gloeosporioides* conidial cells treated with AtTCP21 or melittin (IC_50_ concentration) for 12 h were examined for changes in cell morphology by SEM. Conidial cells treated with AtTCP21 at the MIC displayed notably increased cell surfaces and irregularly sized perforations across the cell exterior (Figure 10). The AtTCP21-treated cells had larger perforations than the melittin-treated cells. Based on these data, it appears that the alterations in morphology can be attributed to the physical damage induced by AtTCP21.

## 4. Conclusions

In summary, the Arabidopsis TCP transcription factor AtTCP21 impedes fungal growth by generating cellular ROS and mitochondrial SOX, thereby inducing apoptosis. This protein is pivotal in the defense mechanism of Arabidopsis against fungal pathogens and is a promising antifungal agent. However, further investigations are warranted to explore its suitability for clinical application. 

## Figures and Tables

**Table 1:** Antifungal activity of AtTCP21 protein against various fungi.

Fungi	IC_50_ (μg/mL)	
	AtTCP21	Melittin
Mold		
*C. gloeosporioides*	3.1	12.5
*F. graminearum*	25	12.5
*F. moniliforme*	50	25
*F. oxysporum*	25	12.5
*F. solani*	25	12.5
*T. harzianum*	25	25
*T. viride*	50	25
Yeast		
*C. albicans*	100	6.3
*C. tropicalis*	100	12.5
*T. beigelii*	50	6.3

## Data Availability

Not applicable.
